# ELDER ABUSE RISK ASSESSMENT MODEL FOR ALZHEIMER’S DISEASE WITH SOCIAL DETERMINANTS OF HEALTH AND MEDICAL FACTORS

**DOI:** 10.1093/geroni/igad104.3297

**Published:** 2023-12-21

**Authors:** Monique Pappadis, Leila Wood, Jordan Westra, Karen Schlag, Rebecca Czyz, Yong-Fang Kuo, Mukaila Raji, Charles Mouton

**Affiliations:** University of Texas Medical Branch at Galveston, Galveston, Texas, United States; University of Texas Medical Branch at Galveston, Galveston, Texas, United States; University of Texas Medical Branch at Galveston, Galveston, Texas, United States; University of Texas Medical Branch at Galveston, Galveston, Texas, United States; University of Texas Medical Branch at Galveston, Galveston, Texas, United States; University of Texas Medical Branch at Galveston, Galveston, Texas, United States; University of Texas Medical Branch at Galveston, Galveston, Texas, United States; University of Texas Medical Branch at Galveston, Galveston, Texas, United States

## Abstract

Older adults with Alzheimer’s Disease and Related Dementias (ADRD) are at an increased risk for elder abuse (EA). Using a 20% national Medicare database, we developed an EA risk-assessment model, including older adults with and without ADRD. Beneficiaries aged 66 and older in 2016, without an EA diagnosis in 2015 or 2016, and Medicare Part A/B/D eligible with no HMO through 2018 were included (N=2,261,166; ADRD, n=187,805, 8.3%). Outcome of interest was an EA diagnosis. The cohort was split into three subsets, training (50%), testing (25%), and validation (25%). Predictive models included demographics, comorbidities, symptoms, injury history, claims-based frailty scores (0-1), medical screening and procedures, and social determinants of health (SDoH). Only 0.2% had an EA diagnosis. EA models were conducted using logistic regression and machine learning methods (random forest, gradient-boosted tree classification, and multilayer perceptron classification). The logistic regression model was the best predictive model (AUC=0.73; Sensitivity=0.80; Specificity=0.47; GINI Impurity=0.50; Agreement=46.77%). Many SDoHs (e.g., primary support and marital problems, housing/income problems and dual eligibility, Black Race), STI testing, behavioral health and medical conditions, and greater frailty were associated with increased risk of EA at 2 years for the total sample (with and without ADRD). For those with ADRD, SDoHs (e.g., marital and housing/income problems), male sex, aged 71-75 (vs 66-70), lung cancer, PTSD , and greater frailty were associated with increased EA risk. Coupled with qualitative interviews, our findings will guide the selection of the most pertinent risk and protective factors to incorporate into an EA screening intervention tool.

